# Cocoon Silk-Derived, Hierarchically Porous Carbon as Anode for Highly Robust Potassium-Ion Hybrid Capacitors

**DOI:** 10.1007/s40820-020-00454-w

**Published:** 2020-05-22

**Authors:** Haiyan Luo, Maoxin Chen, Jinhui Cao, Meng Zhang, Shan Tan, Lei Wang, Jiang Zhong, Hongli Deng, Jian Zhu, Bingan Lu

**Affiliations:** 1 State Key Laboratory for Chemo/Biosensing and Chemometrics, Hunan University, Changsha, 410082 People’s Republic of China; 2 College of Chemistry and Chemical Engineering, Hunan Key Laboratory of Two-Dimensional Materials, Hunan University, Changsha, 410082 People’s Republic of China

**Keywords:** Potassium-ion hybrid capacitors, Biomimetic materials engineering, N-doped carbon, Hierarchically porous structure, High energy density

## Abstract

Potassium-ion hybrid capacitors (KIHCs) have attracted increasing research interest because of the virtues of potassium-ion batteries and supercapacitors. The development of KIHCs is subject to the investigation of applicable K^+^ storage materials which are able to accommodate the relatively large size and high activity of potassium. Here, we report a cocoon silk chemistry strategy to synthesize a hierarchically porous nitrogen-doped carbon (SHPNC). The as-prepared SHPNC with high surface area and rich N-doping not only offers highly efficient channels for the fast transport of electrons and K ions during cycling, but also provides sufficient void space to relieve volume expansion of electrode and improves its stability. Therefore, KIHCs with SHPNC anode and activated carbon cathode afford high energy of 135 Wh kg^−1^ (calculated based on the total mass of anode and cathode), long lifespan, and ultrafast charge/slow discharge performance. This study defines that the KIHCs show great application prospect in the field of high-performance energy storage devices.

**Electronic supplementary material:**

The online version of this article (10.1007/s40820-020-00454-w) contains supplementary material, which is available to authorized users.

## Highlights

- The hierarchically porous nitrogen-doped carbon (SHPNC) was fabricated by biorenewable carbon sources.
- The SHPNC electrode exhibited a high specific capacity, excellent cyclic stability, and superior rate capability.
- The asymmetric potassium-ion hybrid capacitors delivered a maximum energy density of 135 Wh kg^−1^, long lifespan with excellent capacity retention, and outstanding ultrafast charge/slow discharge performance.

**Electronic supplementary material:**

The online version of this article (10.1007/s40820-020-00454-w) contains supplementary material, which is available to authorized users.

## Graphical abstract

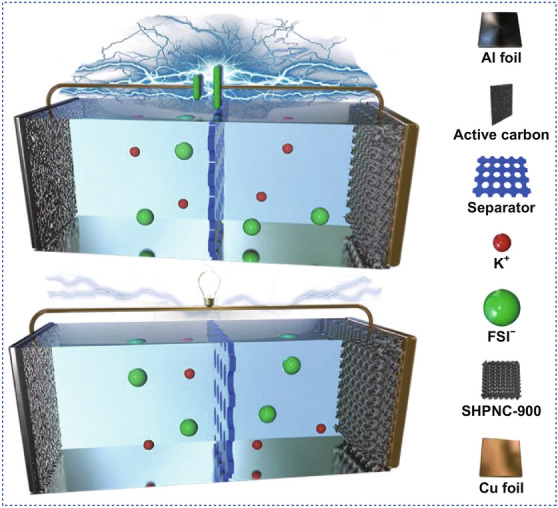

## Introduction

Lithium-ion hybrid capacitors with a battery-type anode and a capacitor-type cathode have been rapidly developed owing to high energy, remarkable power, and long life [1–5]. However, the rising costs and restricted global lithium resources have driven researchers to seek other energy storage devices to relieve the energy crisis [6–8].

Gradually, potassium-ion hybrid capacitors (KIHCs) bridging the gap between potassium-ion batteries (PIBs) and supercapacitors (SCs) are emerging as an exciting research frontier owning to low redox potential, abundant reserves of K (2.09 wt%), higher transport number of solvated K^+^ and lower desolvation energy than that of Li^+^ and Na^+^ [9–12]. Nevertheless, larger ionic radius of the K^+^ (1.38 Å) [13], in sharp contrast to that of Li^+^ (0.76 Å) and Na^+^ (1.02 Å) [14, 15], causes a large volume expansion of the active material during the charging/discharging process, leading to low reversible capacity and inferior cyclic stability as well as insufficient rate capability [16–19]. To improve KIHCs, one efficacious strategy is exploring feasible and sustainable anode materials that can store the large-size K^+^ [13, 17–22]. In contrast with variety of cathode materials reported, searching for high-performance anode materials is much slower and tougher because of unsatisfactory cyclic performance and limited rate capability caused by serious structural deformation, low intercalation utility, and electrolyte decomposition [23]. To date, only limited amount of anode materials have been proposed, such as carbonaceous materials, organic materials, K-ion intercalation compounds, and metal-based alloy materials [24–28]. Especially, the inexpensive carbonaceous materials have been widely studied as one of the leading candidates due to their high thermal stability, high potassium storage capability, and environmental friendliness [29–32].

Biorenewable carbon sources relying on the renewable and widely available advantages have been used as precursors to prepare anode materials for PIBs [33–39]. Cocoon silk with hierarchical architecture featuring an intricate 3D hierarchical network not only ensures extraordinary structural stability, but also allows efficient transport of electrolytes throughout the entire cocoon silk-derived biological carbon matrix [40, 41].

Herein, hierarchically porous nitrogen-doped carbon (SHPNC) was synthesized by a cocoon silk chemistry strategy as an advanced anode material for KIHCs. The SHPNC with highly hierarchical structure and high-content nitrogen doping provides fast pathways of electrons and ions, also offers sufficient free space to overcome the damage caused by the volume expansion during charge and discharge processes. Remarkably, KIHCs were constructed with SHPNC-900 as the battery-type anode and commercial activated carbon (AC) as the capacitor-type cathode. The optimized KIHCs displayed a high energy of 135 Wh kg^−1^, an energy density of 45 Wh kg^−1^ at a high power output of 1951.8 W kg^−1^, and an outstanding cyclic life with the capacity retention of 75.4% after 3750 cycles at 1 A g^−1^.

## Experiment Section

### Synthesis of SHPNC

Synthesis of the SHPNC samples: Firstly, 3 g natural silk and 7.5 g ZnCl_2_ were added to a 2.5 M (50 mL) FeCl_3_ solution. And then, the mixture was stirred and evaporated at 85 °C for 7 h. After the partial solubility of the silk, the mixture was left in drying oven at 95 °C overnight. Then, the as-obtained solution was freeze-dried for 3 days to prepare the carbon precursor. Before activation and graphitization, the precursor was rapidly heated at 150 °C for 1 h with a heating rate of 5 °C min^−1^ to remove the absorbed moisture. Then, the precursor was carbonized at various temperatures (750, 900 and 1050 °C) for 1 h under vacuum environment in a tubular furnace with a heating rate of 2 °C min^−1^. The resulting dark solid was milled, poured into a 1 M HCl solution to soak out the poison iron species and then washed with deionized water. The final obtained porous carbon was dried at 65 °C for 12 h and marked as SHPNC-750, SHPNC-900, and SHPNC-1050, respectively.

### Materials Characterization

The morphology of the samples was characterized via SIGMA microscope (Zeiss, Germany) and transmission electron microscope (TEM, 2100F, JEOL). The crystal structures were explored by X-ray diffractometer (XRD-6100 spectrometer with Cu-Kα radiation, Shimadzu) and Raman spectrometer (inVia-reflex confocal Raman spectrometer, Renishaw) with a 532 nm laser as the excitation source. XPS spectra were obtained on a K-Alpha ESCALAB 250Xi instrument (ThermoFisher-VG Scientific, USA), with Al Kα radiation as the excitation source. N_2_ adsorption–desorption analysis was measured on surface area and porosity analyzer (ASAP 2020, Micromeritics). BET method and the Barrett–Joyner–Halenda (BJH) method were performed to deduce the specific surface area and pore size distribution.

### Electrochemical Measurements

SHPNC, conductive carbon, and carboxymethyl cellulose with mass ratio of 8:1:1 were dispersed in a mixed solution (1 mL) of ethanol and H_2_O and painted on the Cu foil after ball-milling treatment. The mass loading of SHPNC electrode is about 0.5–2 mg cm^−2^. Besides, the active carbon, conductive carbon, and carboxymethyl cellulose were mixed together with a weight ratio of 8:1:1 to prepare the cathode. The mass ratio of anode and cathode was 3:1. The electrodes were nature dried for 1 h followed by vacuum drying at 60 °C overnight. After drying at 120 °C under vacuum, 2032-type coin cells were fabricated inside an Ar filled glovebox, employing a glass fiber filter (Whatman GF/F) as the separator and 5 M KFSI dissolved in ethylene carbonate/dimethyl carbonate mixture (EC/DMC by 1:1 vol.) as electrolyte. To assemble PIBs, potassium metal and glass fiber film were used as the anode electrode and the separator, respectively. The coin-type cells (2032) were assembled in a MB-Labstar (1200/780) glove box (Munich, Germany) under Ar atmosphere, where the concentrations of moisture and oxygen were maintained below 0.5 ppm. The electrochemical performance and cyclic voltammetry (CV) were tested using a CT2001A battery test system (LANDTE Co., China) and a CHI660E electrochemical station (CHI instrument Co., Shanghai, China), respectively.

The gravimetric energy (*E*) and gravimetric power (*P*) of devices were calculated according to the following equations:

1 $$E = \mathop \smallint \limits_{t1}^{t2} IU/m{\text{d}}t$$

2 $$P = E/t$$

where *m* is the total mass of the both electrodes, *U* is the working voltage, *I* is the discharge current and *t* is the discharge time at the end of discharge after the IR drop.

## Results and Discussion

### Preparation and Structure of SHPNC

**Fig. 1:**
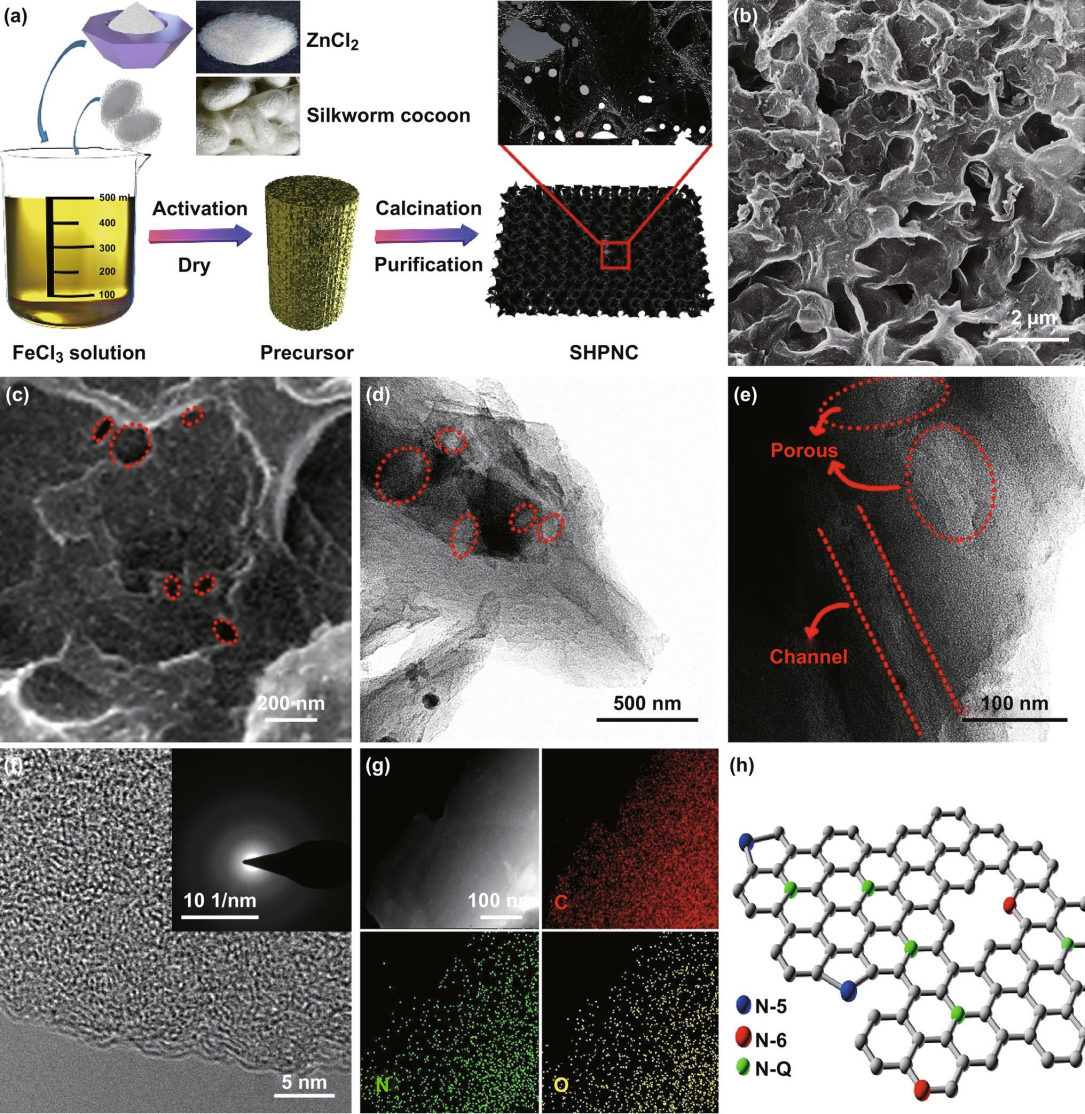
**a** Schematic illustration of the preparation process for the SHPNC. Structure characterizations of SHPNC-900 by electron microscopies. **b**, **c** SEM and **d**, **e** TEM images of SHPNC-900. **f** HRTEM images of SHPNC-900 with inset showing the corresponding SAED patterns. **g** Corresponding elemental mapping images of C, N, and O elements of the SHPNC-900. **h** Corresponding porous structure diagram

Figure 1 reveals the schematic illustration of synthetic process, morphology, and composition of the SHPNC calcined at 900 °C (SHPNC-900). As shown in Fig. 1a, the cocoon silk chemistry strategy to synthesize SHPNC consists of four steps: (i) metal salt activation, (ii) freeze drying, (iii) calcination, and (iv) purification process. The resulting unique SHPNC possesses prominent features of high specific surface area with abundant active sites through hierarchical carbon matrix, rich heteroatom doping, and defects [42–45]. As shown in scanning electron microscopy (SEM) in Fig. 1b, c, the as-obtained SHPNC shows a highly porous structure composed of interconnected and wrinkled carbon nanosheets, which effectively limit them from stacking together. The superior structural feature plays important roles in enhancing the transportation of electrons/potassium-ions, and affording free expansion space for electrode [46]. Transmission electron microscopy (TEM) images (Figs. 1d, e and S1) further indicate the SHPNC is formed of folded, porous, and multichannel carbon nanosheets [47]. Besides, no apparent long-range ordered areas can be found in the SHPNC, revealing an amorphous structure of SHPNC. Furthermore, the selected-area electron diffraction (SAED) pattern (inset of Fig. 1f) presents dispersed diffraction rings, which is well consistent with the HRTEM results (Fig. 1f). As clearly observed in element mapping images (Figs. 1g and S1), the C, O, N elements were uniformly distributed over the entire nanosheets and the content of N significantly reduced with the annealing temperature increasing. In addition, the corresponding porous structure diagram is presented in Fig. 1h. Notably, the N doped in the SHPNC-900 divided into pyridinic nitrogen (N-6), pyrrolic or pyridonic nitrogen (N-5), and quaternary nitrogen (N-Q), respectively, can effectively improve the conductivity and increase the electrochemical active sites of the SHPNC-900 electrode [48].

**Fig. 2:**
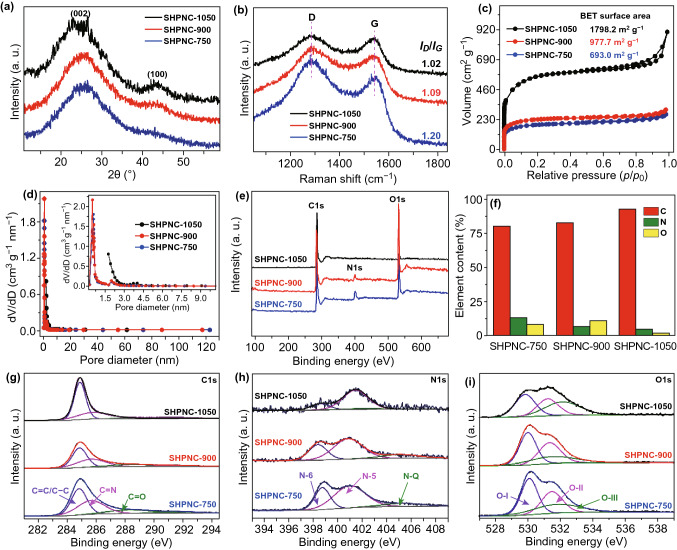
Structure characterizations of SHPNC by spectroscopies. **a** XRD patterns. **b** Raman spectra. **c** Nitrogen adsorption–desorption isotherms. **d** Pore size distribution. **e** XPS survey spectra of SHPNC. **f** Specific content of C, O, and N in SHPNC. **g** C 1 s, **h** N 1 s and **i** O 1 s core level XPS high-resolution spectra

X-ray diffraction (XRD) and Raman spectroscopy measurements are conducted to characterize the structures of SHPNC annealed at 750, 900, and 1050 °C (SHPNC-750, SHPNC-900, and SHPNC-1050). It aims at verifying whether SHPNC processes a high degree of local order or much disorder in nanometric scales. As shown in Fig. 2a, all the three samples show broad diffraction peak at about 25° and another weak diffraction peak at about 44°, corresponding to the (002) and (100) planes of the graphite, respectively. With increasing annealing temperature, the (100) peak becomes relatively sharp, indicating minified interlayer spacing of the (100) plane [48]. Raman spectra of SHPNC-750, SHPNC-900, SHPNC-1050 in Fig. 2b exhibit the D and G bands appearing around 1337 and 1540 cm^−1^. The D band stands for defects induced *A*_1g_ vibration mode of *sp*^3^ carbon rings, while the G band stands for *E*_2g_ vibration mode of *sp*^2^ carbon atoms [49]. Additionally, the intensity ratio of D band and G band (*I*_D_*/I*_G_) can be used to denote degree of disorder in the graphite [50]. Obviously, the degree of graphitization increases as the rise of annealing temperature.

The porosity and architecture of the as-fabricated SHPNC were verified by nitrogen absorption–desorption isotherms and pore size distribution analyses. Figure 2c shows a high nitrogen uptake at lower pressure caused by micropores and the hysteresis loop at higher pressure due to mesopores. All SHPNC samples exhibit type I isotherms and the high isotherm in the lower pressure region, suggesting a high microporosity. Brunauer–Emmett–Teller (BET) surface areas are calculated to be 693.0 (SHPNC-750), 977.7 (SHPNC-900), and 1798.2 m^2^ g^−1^ (SHPNC-1050). As shown in Fig. 2d, the pore sizes of three samples are concentrated between 0 and 3 nm, which is consist with the adsorption–desorption isotherm.

In order to quantify the chemical composition of the SHPNC, surface characteristics were analyzed by X-ray photoelectron spectroscopy (XPS). As displayed in Fig. 2e, two sharp peaks indicate SHPNC-1050 is mainly composed of C and O, while three peaks in SHPNC-750 and SHPNC-900 demonstrate the as-prepared SHPNC-750 and SHPNC-900 are mainly composed of C, N, and O. The specific element contents of C, N, and O are listed in Fig. 2f, implying the contents of N element decreases as the rise of annealing temperature, which agrees with the result of TEM element mapping. Besides, the high-resolution C1s core level XPS spectra of SHPNC shown in Fig. 2g deconvolute to three peaks (C–C (284.8 eV), C=N (285.7 eV), and C=O (288.7 eV)), indicating the synthetic strategy is a powerful to in situ synthesize heteroatom doping SHPNC [51, 52]. The N 1 s spectra (Fig. 2h) reveals three similar peaks for all samples at binding energies of 398.7, 400.9, and 405.1 eV, which are indexed to N-6, N-5, and N-Q, respectively. The content of N-6 relative to N-Q reduces with increasing temperature, indicating that higher calcination temperature facilitates the generation of N-Q (Fig. S2). It is noteworthy that N-6 and N-5 can act as electrochemically active sites and be beneficial to the surface-induced capacitive processes [40]. Meanwhile, N-Q is reported to be conducive to the electroconductibility of the graphitic carbon relying on the significant change in the electron–donor characteristic [53]. Figure 2i shows that three similar peaks of O 1 s spectrum at about 530.0, 531.4, and 531.6 eV can be indexed to C=O carbonyl groups (O–I), C–OH hydroxylic groups or C–O–C ether groups (O–II), and COOH carboxyl groups (O–III), respectively [52].

### Electrochemical Properties of SHPNC Electrode for Potassium-Ion Half Cells

**Fig. 3:**
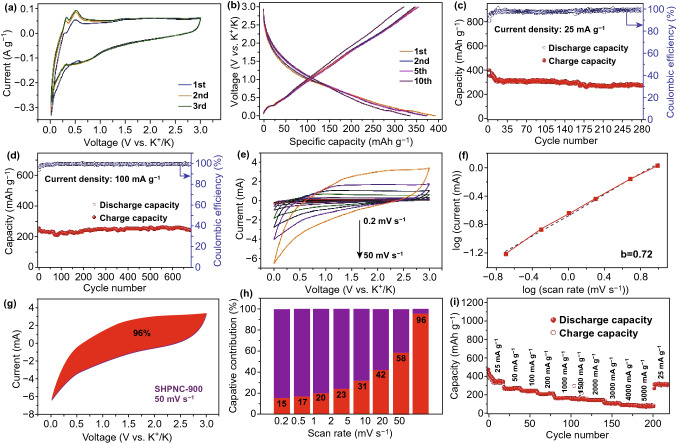
Electrochemical performance of SHPNC-900 as PIB anode in half cells. **a** Cyclic voltammograms (CV) for the first three cycles of SHPNC-900. **b** Charge–discharge voltage profiles for selected cycles of SHPNC-900 composite at a current density of 25 mA g^−1^. Cycling performance of SHPNC-900 at a current density of **c** 25 and **d** 100 mA g^−1^. **e** CV curves of SHPNC-900 at various scan rates of 0.2 to 50 mV s^−1^. **f** b value determination. **g** Contribution of the surface process at scan rate of 20 mV s^−1^ in SHPNC-900. **h** Contribution of the surface process in the SHPNC-900 at different scan rates. **i** Rate performance of SHPNC-900 with rates ranging from 25 to 5000 mA g^−1^

Cyclic voltammetry (CV) was conducted to investigate electrochemical reaction of SHPNC (Figs. 3a and S3). The CV curves of these three samples are similar, while the SHPNC-900 displays larger peak area due to its higher specific capacity. Figure 3a shows the first three consecutive cyclic voltammograms of the SHPNC-900 electrode at a scan rate of 0.2 mV s^−1^ in the voltage range of 0.01–3 V. There are three peaks in the first cycle including a clear anodic peak at 0.499 V, a relatively weak peak at 0.304 V, as well as a cathodic peak at 0.578 V. The cathodic peak ascribed to the formation of solid electrolyte interface (SEI) and the decomposition of the electrolyte, weakens in the subsequent scans [53]. Accordingly, the anodic peak at 0.304 V could be attributed to the deintercalation of K ions [52]. Obviously, the following two CV curves exhibit the similar characteristics, demonstrating the same reaction mechanism and good reversibility of SHPNC-900 anode. Figure 3b presents the charge/discharge voltage profiles of the SHPNC-900 anode in selected cycles at a current density of 25 mA g^−1^ in the voltage range of 0.01–3.0 V (vs. K/K^+^). Apparently, the different cycles show similar potassiation/depotassiation behavior. Equally, the charge/discharge profiles almost overlap with each other (Fig. S4a-c), which is consistent with the CV results, indicating a good reversibility of SHPNC electrode. In addition, with increasing current density, the platform at ≈ 0.2 V almost disappears while the charging process and the linear characteristic of the discharge/charge curves becomes more obvious (Fig. S4d).

Electrochemical performance of as-prepared three samples (SHPNC-750, SHPNC-900, and SHPNC-1050) was investigated to explore the influence of carbonization temperature. Figure S5 is the charge–discharge voltage profiles for selected cycles of SHPNC at a current density 500 mA g^−1^, demonstrating similar battery behavior of SHPNC-750, SHPNC-900, and SHPNC-1050 electrodes. Figure S6 shows that the SHPNC-900 displays best cycling stability and possesses the highest reversible capacity of 215 mAh g^−1^ at 500 mA g^−1^ after continuous 370 cycles. Notably, the SHPNC-900 with appropriate specific surface area and nitrogen content (N-5, N-6, and N-Q) compared with that of SHPNC-750 and SHPNC-1050 (Fig. S2) achieves continuous higher capacity and superior cyclic performance. Thus, we mainly focus on investigations of the battery performance of SHPNC-900 electrode.

As shown in Fig. 3c, the SHPNC-900 delivers a high reversible capacity of 300 mAh g^−1^ at current density of 25 mA g^−1^ after 163 cycles. At current density of 200 mA g^−1^, the SHPNC-900 electrode displays a high reversible capacity of 270 mAh g^−1^ after 923 cycles (Fig. 3d). Furthermore, long-term cyclability of SHPNC-900 was investigated at other current rates (Fig. S7). As expected, it exhibited impressive cycling stability with high specific capacities of 271 (50 mA g^−1^) and 164 mA h g^−1^ (1000 mA g^−1^), after 330 and 593 cycles, respectively. Remarkably, the electrochemical performance of SHPNC is superior compared to that of other reported carbon-based materials due to the unique cocoon silk chemistry strategy [21, 25, 46, 49, 50, 53–57].

As displayed in Fig. 3e, CV curves were measured at scan rates of 0.2 to 50 mV s^−1^ in a voltage range from 0.01 to 3 V to analyze the kinetic of the electrodes. The capacity contribution in the SHPNC-900 electrode was examined in details according to the power-law formula *i *= *aν*^*b*^ [58, 59], where *i* is the peak current and *ν* is the scan rate. Clearly, the b value can be obtained by the slope of the log(*i*) versus log(*υ*) plot. When the b value is close to 0.5, the electrochemical behavior is predominated by the ionic diffusion process, while the b value close to 1.0 indicates a total capacitive process [60, 61]. And the plot applied on the depotassiation peak current is shown in Fig. 3f. A good linear relationship can be seen for SHPNC-900, and the b value was calculated to be 0.72, suggesting a mixed potassium storage mechanism of Faradaic intercalation process and surface process. To be specific, the equation of *i *=* k*_*1*_*v *+* k*_*2*_*v*^*1/2*^ can quantify the capacitive contribution ratio under different scan rates, where *k*_*1*_*v* and *k*_*2*_*v*^*1/2*^ represent the contribution of capacitance and ionic diffusion, respectively [58, 59]. A typical profile (Fig. 3g) at a scan rate of 50 mV s^−1^ manifests the separation of the capacitive contribution (red region) from the total capacity (purple region). The capacitive capacity of SHPNC-900 accounts for 15% at a low scan rate of 0.2 mV s^−1^, indicating that the charge storage behavior is dominated by the ionic diffusion process (Fig. S8a). As the scan rate rising to 0.5, 1, 5, 10, 20, and 50 mV s^−1^, the fraction of capacitive capacity increases to 17%, 20%, 31%, 42%, 58%, and 96%, respectively (Figs. 3h and S8). This phenomenon further confirmed that SHPNC-900 assists PIBs with superior rate capability. Rate performance is important in practical applications of PIBs in electric vehicles and power tools. Thus, capacities versus cycle number at various charge/discharge current rates were investigated over a voltage range of 0.01–3.00 V versus K/K^+^ (Fig. 3i). Noticeably, the SHPNC-900 electrode delivers high discharge capacities of 343, 273, 245, 216, 168, 152, 145, 107, 89, and 78 mAh g^−1^ at the current densities of 25, 50, 100, 200, 1000, 1500, 2000, 3000, 4000, and 5000 mA g^−1^, respectively. Moreover, when the current density returns to 25 mA g^−1^, about 93% of the discharge capacity can be recovered (a reversible capacity of 318 mAh g^−1^ obtained after 200 cycles), revealing a superior rate stability of SHPNC-900. The hierarchically porous nitrogen-doped SHPNC-900 provides outstanding structural stability, fast ion, and electron transport during the charge/discharge process, as well as strong pseudocapacitive behavior, thus affording high utilization and rate capability of SHPNC-900 electrode [40].

### Reversibility Analysis and Mechanism Exploration of SHPNC-900 Electrode

**Fig. 4:**
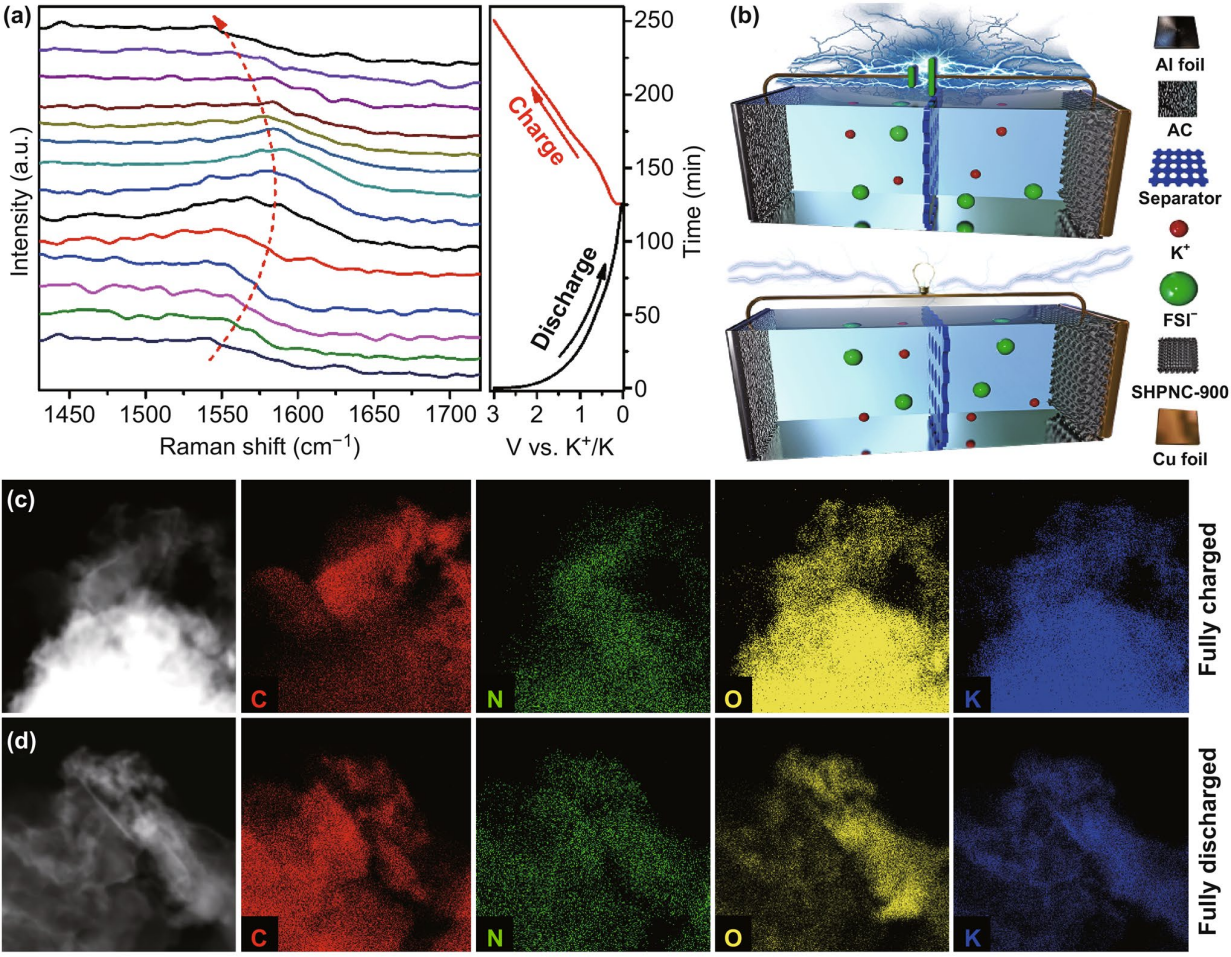
**a** In situ Raman spectra of SHNPC-900 during potassiation/depotassiation in potassium half cells. **b** Schematic illustration of the charge/discharge mechanism of the KIHC. **c**, **d** Element mapping of the SC anode in KIHCs at different states: **c** charged state, **d** discharged state

In situ Raman spectroscopy and element mapping were systematically carried out to visualize the potassiation/depotassiation mechanism of SHPNC-900 electrodes (Fig. 4). As shown in Fig. 4a, the initial G band (1539 cm^−1^) obviously blue shifts to lager values and reaches 1591 cm^−1^ at voltage of 0.01 V during discharge process. Upon full charge (3 V), the G band shifted toward the lower wave number until it recovers to the original value (1540 cm^−1^), which signifies that a reversible reaction occurs. To evaluate the feasibility of the as-obtained SHPNC for practical application, full KIHCs were assembled employing activated carbon (AC) as cathode, SHPNC-900 as anode, and 5 M KFSI dissolved in ethylene carbonate/dimethyl carbonate mixture (EC/DMC by 1:1 vol.) as electrolyte. It is worth noting that, superconcentrated KFSI exhibits better thermodynamic reactivity and improves the formation of KF-rich SEI to suppress the electrolyte decomposition and the formation of K dendrite formation. Therefore, 5 M KFSI electrolyte enables preferable reversible capacity and better cycling stability of KIHCs [62–66]. The schematic illustration of the configuration of KIHCs with SHPNC-900 anode, commercial AC cathode, and a 5 M KFSI electrolyte is illustrated in Fig. 4b. Upon charging/discharge, the anions (FSI^−^) in the electrolyte adsorb on the AC cathode, while the cations (K^+^) intercalate into anode materials. The discharge process is reversible to the charge process. Elemental mapping was performed to further verify the working mechanism of the KIHCs (Fig. 4c, d). The fully charged and fully discharged states of SHPNC-900 anode are composed of C, N, O, and K elements. Obviously, a large number of K^+^ are detected in the fully charged state, while the content of K^+^ in fully discharged is much lower, which implies that K^+^ is able to reversibly intercalate/de-intercalate into SHPNC-900 anode. Based on in situ Raman and element mapping, the satisfactory reversibility in the charge/discharge process can be a prerequisite for superior cyclic stability.

### Electrochemical Properties of SHPNC-900 Electrode for K-Ion Hybrid Capacitors

**Fig. 5:**
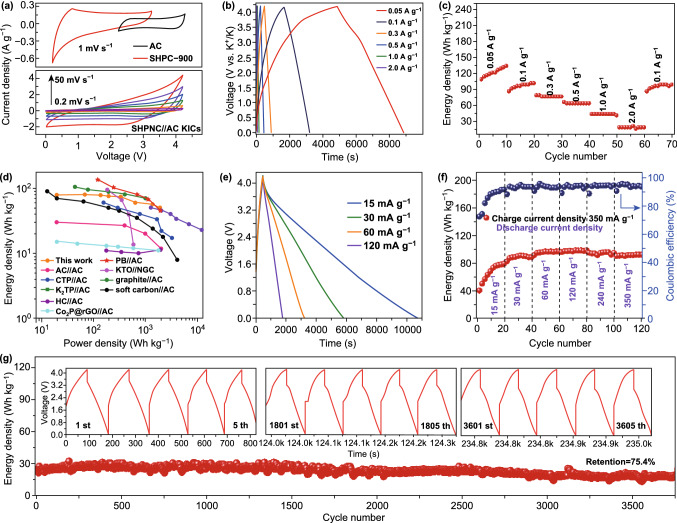
Electrochemical properties of SHPNC-900 electrode for K-ion hybrid capacitors. **a** CV curves of SHPNC and AC in half cells (top) and full cell of KIHC (bottom). **b** Profile of the charge–discharge curves for full capacitors at different current densities. **c** Rate performance of the KIHC. **d** Ragone plot of SHNPC//AC compared with other energy storage devices and another KIHC device. **e** Ultrafast charge/slow discharge profiles of KIHC with a constant charging at 350 mA g^−1^ while being discharged at various current densities. **f** KIHC being charged at 350 mA g^−1^ and discharged at various current densities. **g** Long-cycle performance at a current density of 1 A g^−1^. Inset: charge–discharge curves

CV tests of SHPNC-900 anode and AC cathode are performed to match KIHCs as shown at the top of Fig. 5a. Additionally, CV curves of the KIHCs at various sweeping speeds from 0.2 to 50 mV s^−1^ in a voltage range from 0.01 to 4.2 V are exhibited at the bottom. All CV curves show semblable rectangular shapes without obvious redox peaks, indicating a capacitive-dominant behavior [67, 68]. With increasing of scan rates, CV curves still keep alike characteristics without a significant distortion, displaying a benign reversibility [69]. Figure 5b exhibits the measured galvanostatic charge–discharge profiles ranging from 0.05 to 2 A g^−1^. It is worth mentioning that the KIHCs could operate for almost 9000 s at 50 mA g^−1^. Figure 5c shows the KIHCs deliver high energy density of 135, 102, 77.5, 65, 45, 20 Wh kg^−1^ at the current densities of 50, 100, 300, 500, 1000, 2000 mA g^−1^, respectively. The Ragone plot in Fig. 5d (relationship between energy and power densities) of the KIHCs device based on the total mass of two electrodes shows that energy-power characteristics of SHPNC-900//AC are markedly superior to the results for most of previously reported KIHCs including graphite//AC [70], soft carbon//AC [15], K_2_Ti_6_O_13_//AC (KTO//AC) [10], Ca_0.5_Ti_2_(PO_4_)_3_@C//AC (CTP//AC) [11], Co_2_P@rGO//AC [14], cubic Prussian blue//AC (PB//AC) [71], dipotassium terephthalate//AC (K_2_TP//AC) [22], AC//AC [4], and HC//AC [4]. Discharging for a long period of time (slow discharge) while completing a full charge quickly (fast charge) implies an excellent fast charge/slow discharge performance [15]. Figure 5e and S9 show the ultrafast charge/slow discharge characteristics of the KIHCs which is also appraised for applying energy storage device in electronic devices and electric vehicles. Surprisingly, the KIHCs could be fully charged within 7 min at 350 mA g^−1^ and discharge for over 2.5 h at 15 mA g^−1^ (Fig. 5e). The KIHCs are charged to 4.2 V at a constant current density of 350 mA g^−1^ (Fig. 5f) and 500 mA g^−1^ (Fig. S10), respectively, and then discharged to 0.01 V at the current densities of 15, 30, 60, 120, 240, 350, and 500 mA g^−1^, respectively. In initial 20 cycles, the energy density increases should be ascribed to the gradual permeation of electrolyte into the structural interior of SHPNC-900 and AC with high specific surface area. Gradually, it could deliver energy densities of 79.4, 88.9, 97.4, 99.1, 95.1, and 92.6 Wh kg^−1^, displaying the excellent fast charge and slow discharge property. As shown in Fig. 5g, the optimized SHPNC-900//AC KIHCs display outstanding cyclic reversibility and can retain 75.4% of its initial capacity after over 3750 cycles in a voltage window 0.01–4.2 V. Figure S11 is the cycle performance of KHICs at current density of 2 A g^−1^. The energy density of the KIHCs also remains stable over 1858 cycles. The remarkable cycle stability of this KIHCs device can be attributed to the typical hierarchically porous structure of SHPNC and high-content nitrogen doping via cocoon silk chemistry strategy.

## Conclusions

In summary, the cocoon silk-derived, hierarchically porous nitrogen-doped carbon was fabricated via cocoon silk chemistry strategy as anode for highly robust KIHCs. Hierarchically porous structure and high-content nitrogen doping within SHPNC afford fast ion and electron transport, play important roles in buffering volume changes of K-ion, and increase the electrochemical active sites. Thus, the KIHCs with SHPNC anode and activated carbon cathode achieve a high energy density of 135 Wh kg^−1^ at a power density of 112.6 W kg^−1^ and outstanding cyclic stability. Furthermore, an ultrafast charge/slow discharge performance with a full charge in just 7 min and a discharge time of more than 2.5 h demonstrates a practical potential of the SHPNC//AC KIHCs. This work offers a pathway to design biological carbon to be a prospective and effective anode material for functional KIHCs.

## Electronic supplementary material

Below is the link to the electronic supplementary material.

Supplementary material 1 (PDF 1023 kb)
