# Supplementary material for: Cocoon Silk-Derived, Hierarchically Porous Carbon as Anode for Highly Robust Potassium-Ion Hybrid Capacitors
Source: Nanomicro Lett. 2020 May 22;12:113. doi: 10.1007/s40820-020-00454-w (PMC7770764; doi:10.1007/s40820-020-00454-w)
Supplement: Supplementary file 1 — Supplementary material 1 (PDF 1023 kb) [file 40820_2020_454_MOESM1_ESM.pdf]

Supporting Information for

Cocoon Silk Derived, Hierarchically Porous Carbon as Anode for Highly Robust Potassium-Ion Hybrid Capacitors

Haiyan Luo[1, 2], Maoxin Chen[1, 2], Jinhui Cao[1, 2], Meng Zhang[1, 2], Shan Tan[1, 2], Lei Wang[1, 2], Jiang Zhong[1, 2], Hongli Deng[1, 2], Jian Zhu[1, 2, *], Bingan Lu[1, 2]

[1]State Key Laboratory for Chemo/Biosensing and Chemometrics, Hunan University, Changsha 410082, People's Republic of China

[2]College of Chemistry and Chemical Engineering, Hunan Key Laboratory of Two-Dimensional Materials, Hunan University, Changsha 410082, People's Republic of China

*Corresponding author. E-mail: jzhu@hnu.edu.cn (Jian Zhu)

Supplementary Figures

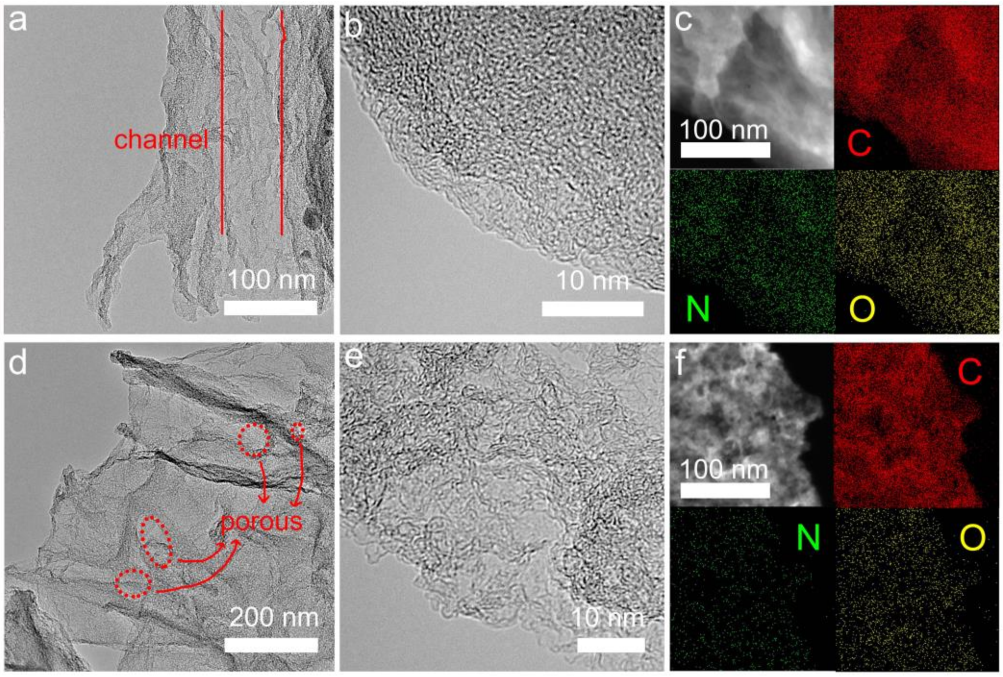

Fig. S1 a TEM images of SHPNC-750. **b** HRTEM images of SHPNC-750. **c** Corresponding elemental mapping images of C, N, and O elements of the SHPNC-750. **d** TEM images of SHPNC-1050. **e** HRTEM images of SHPNC-1050. **f** Corresponding elemental mapping images of C, N, and O elements of the SHPNC-1050

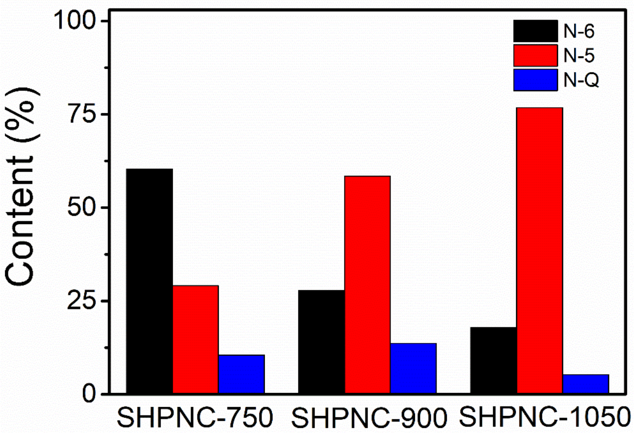

Fig. S2 Specific content of N-6, N-5, and N-Q in SHPNCs

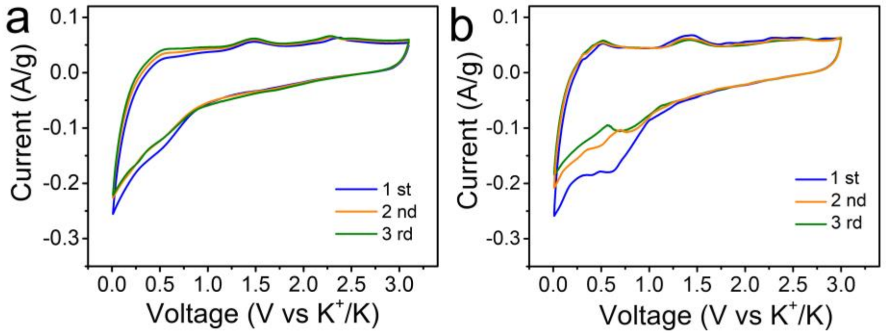

Fig. S3 Cyclic voltammograms (CV) for the first three cycles of **a**) SHPNC-750 and **b**) SHPNC-1050

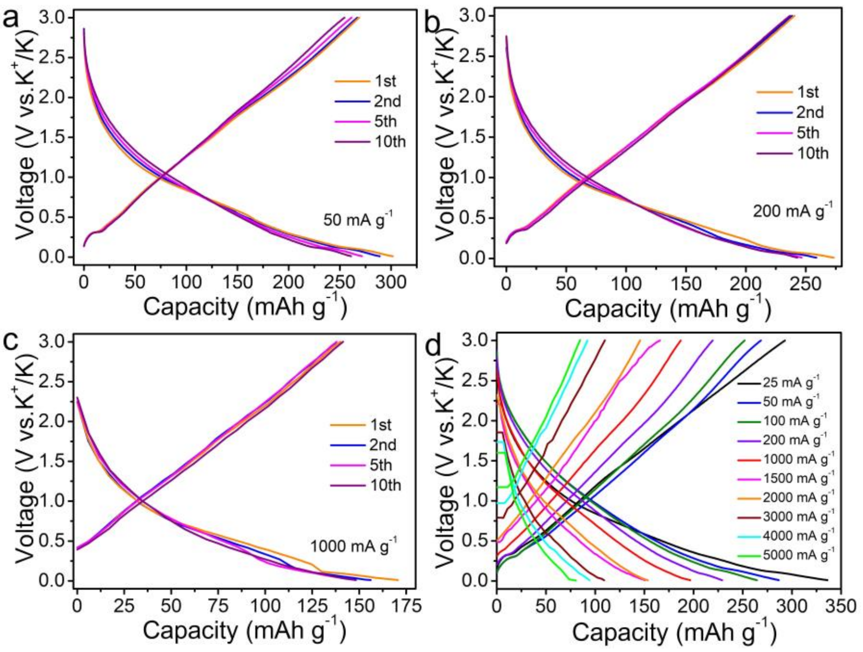

Fig. S4 Charge–discharge voltage profiles for selected cycles of SHPNC-900 at current densities of (**a**) 50, (**b**) 200 and (**c**) 1000, (**d**) Discharge rate capability of SHPNC-900 at current densities from 25 to 5000 mA g^{-1}

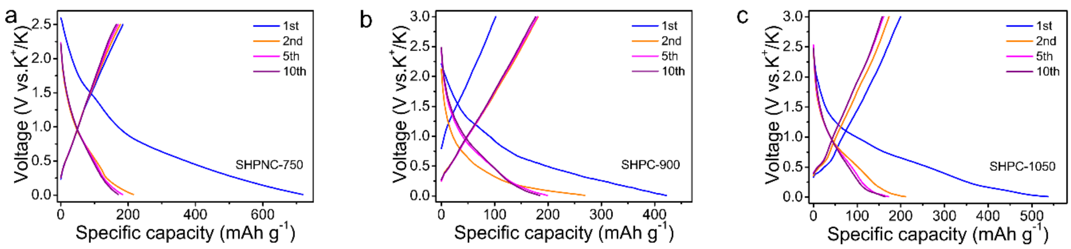

Fig. S5 Charge–discharge voltage profiles for selected cycles of SHPNCs at a current density 500 mA g^{-1}

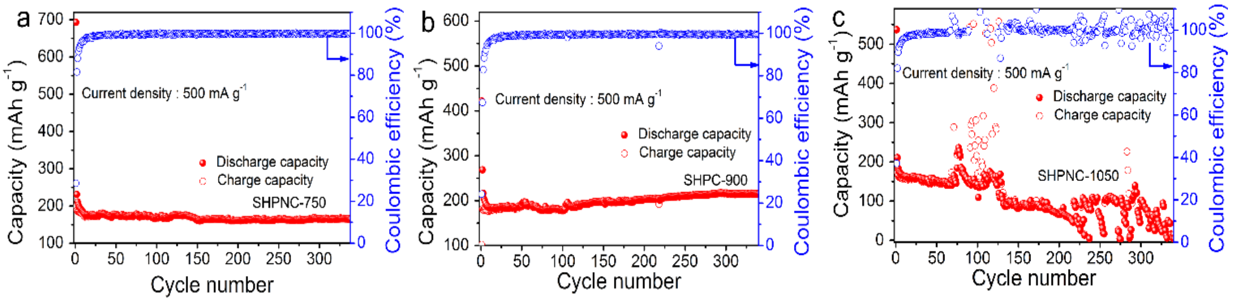

Fig. S6 Comparison of the cycling performance of the (**a**) SHPNC-750, (**b**) SHPNC-900 and (**c**) SHPNC-1050 electrodes at a current density 500 mA g^{-1} for 370 cycles

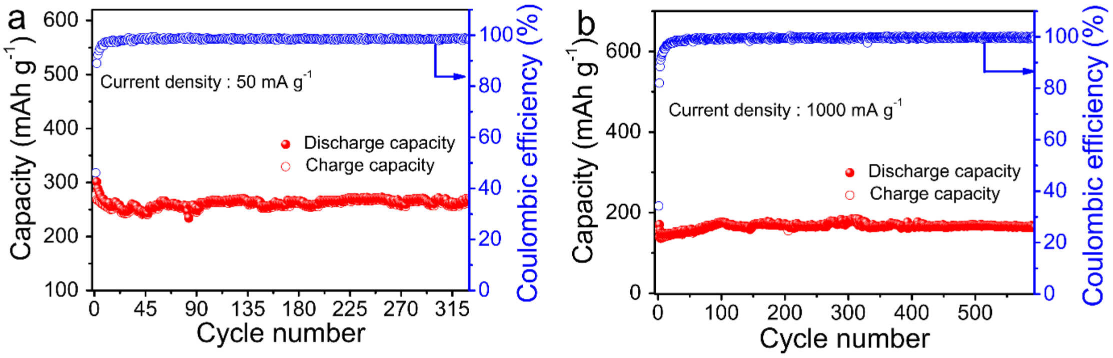

Fig. S7 Cycling performance of SHPNC-900 at a current density of (**a**) 50 and (**b**) 1000 mA g^{-1}

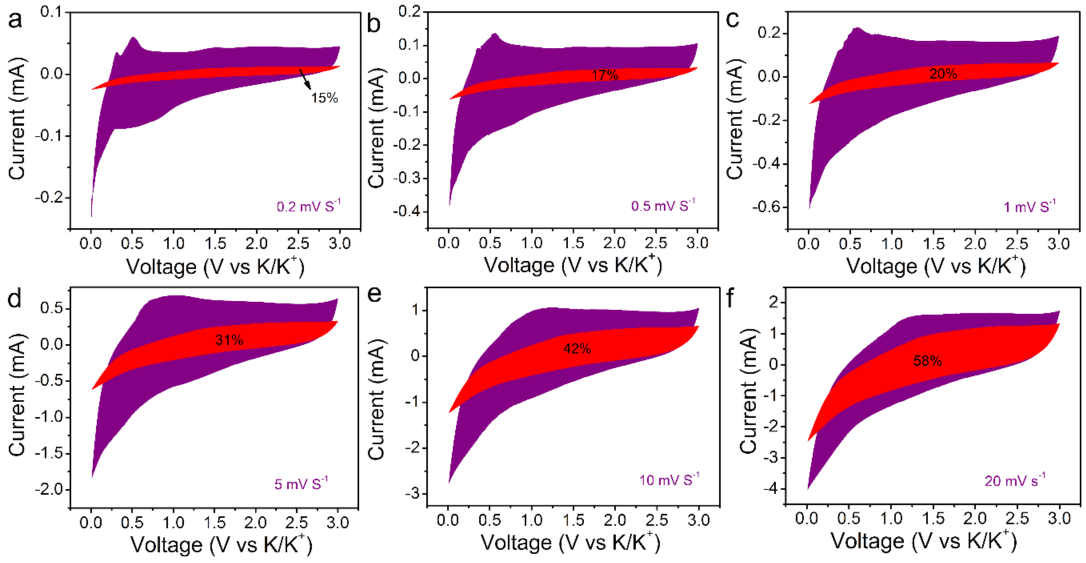

Fig. S8 Contribution of the surface process of SHPNC-900 at scan rate of (**a**) 0.2, (**b**) a 0.5, (**c**) 1, (**d**) 5, (**e**) 10 and (**f**) 20 mV s^{-1}

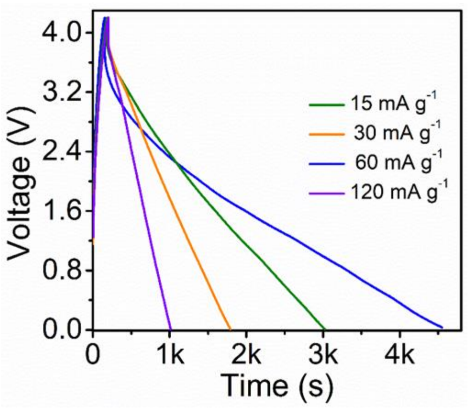

Fig. S9 The ultrafast charge/slow discharge profiles of KIHC with a constant charging at 500 mA g^{-1} while being discharged at various current densities

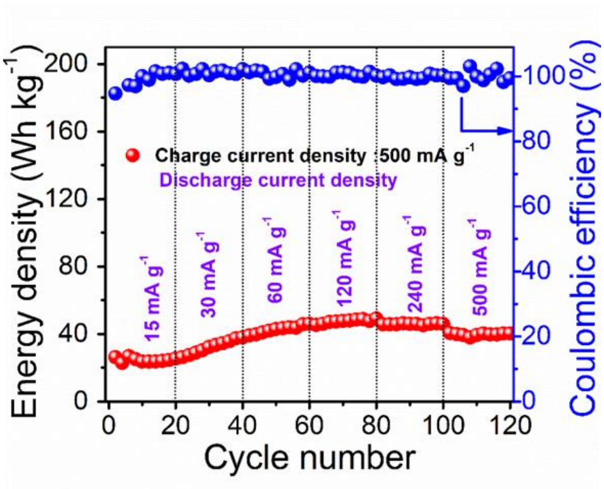

Fig. S10 KIHC being charged at 500 mA g^{-1} and discharged at various current densities

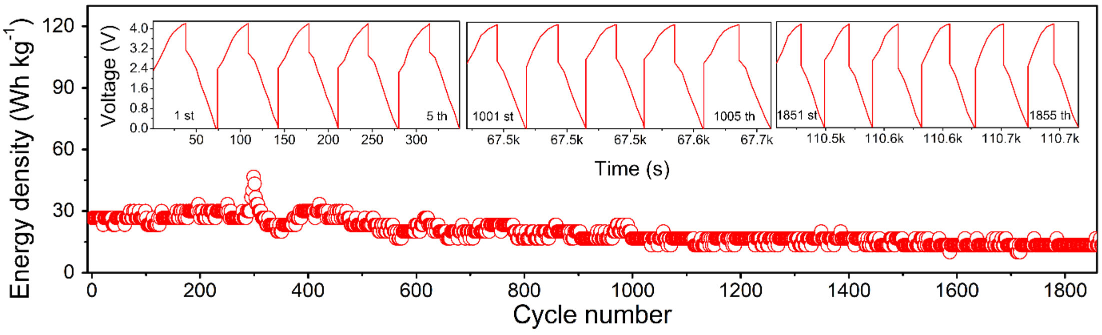

Fig. S11 Long-cycle performance at a current density of 2 A g^{-1}. Inset: charge–discharge curves
